# “It Felt Like Practicing Telemedicine While Learning About It”: A Reflexive Thematic Analysis of Medical Students’ Reflective Diaries from a Telemedicine Elective Course Across two Cohorts

**DOI:** 10.1007/s40670-026-02914-1

**Published:** 2026-10-14

**Authors:** Motti Haimi, Irit Chudner

**Affiliations:** 1 The Ruth and Bruce Faculty of Medicine, Technion – Israel Institute of Technology, Haifa, Israel; 2 Health Systems Management Department, Yezreel Valley College, Afula, Israel; 3 School of Behavioral Sciences, The College of Management Academic Studies, Rishon LeTsiyon, Israel

**Keywords:** Telemedicine, Medical education, Reflective writing, Qualitative research, Thematic analysis, Pre-clinical curriculum, Doctor–patient relationship, Artificial intelligence

## Abstract

**Background:**

Telemedicine has shifted from a niche modality to an established component of routine clinical practice in many health systems. Medical curricula are only beginning to address this shift. Reflective writing is increasingly used both as a pedagogical tool in medical education and as a data source for qualitative research on student attitudes. The experiences and conceptual journeys of pre-clinical medical students enrolled in dedicated telemedicine instruction are under-studied, particularly across multiple cohorts spanning the COVID-19 and post-pandemic periods.

**Methods:**

We conducted a reflexive thematic analysis of 81 reflective diaries written by students (predominantly second-year medical students) enrolled in an elective telemedicine course at a single Israeli medical school across two cohorts distinguished by consent procedure: a retrospective cohort (2019–2024, *n* = 39), in which students who had already submitted diaries as a routine course component were contacted by email in 2025 (after ethical approval was granted) and asked to consent retrospectively to use of their diaries in research; and a prospective cohort (2025, *n* = 42), in which students were informed at the start of the course that the diaries would be analyzed and were asked to enclose a signed consent form with the diary they submitted. Analysis followed Braun and Clarke’s six-phase reflexive thematic analytic framework with an inductive orientation.

**Results:**

Six overarching themes were identified: (1) a transformative conceptual journey from skepticism or naïveté to conviction; (2) telemedicine as the inevitable trajectory of medicine and a site of professional-identity formation; (3) recognition of telemedicine as a holistic discipline drawing on multiple lenses; (4) a productive tension between equity gains and erosion of the doctor–patient bond; (5) lived experience (as patient, family member, or in prior professional roles) functioning as an explicit interpretive lens; and (6) pedagogy and the medium of delivery, with the Zoom format read by students as enacting the course’s argument. The thematic structure was stable across cohorts. Distributional shifts in the prospective (2025) cohort included articulation of artificial-intelligence displacement concerns, focused engagement with breaking-bad-news as a boundary case, named concerns about defensive prescribing, the war (October 2023 onward) as a catalyzing event alongside COVID-19, increased advocacy for converting the course from an elective to a curricular requirement, and emerging attention to online patient and clinician communities as a distinct dimension of digital medicine.

**Conclusions:**

This multi-cohort qualitative analysis identifies six stable themes and documents strong, consistent positive engagement with a pre-clinical telemedicine elective across both cohorts. Students describe the course as a formative and highly valuable learning experience, engage substantively with the multi-dimensional character of telemedicine, and read the Zoom delivery format as enacting the course’s content — an effect we describe as pedagogical resonance. Differences in the articulation of certain sub-themes (notably AI-displacement concerns and humanistic boundary cases such as breaking bad news) were more prominent in the prospective (2025) cohort and reflect the evolving discourse on digital medicine; these are cross-sectional differences and do not warrant longitudinal or causal inference. Taken together, our findings make a strong case for the inclusion of dedicated telemedicine instruction as a core component of the pre-clinical medical curriculum.

**Supplementary Information:**

The online version contains supplementary material available at https://doi.org/10.1007/s40670-026-02914-1.

## Introduction

Telemedicine — the delivery of clinical care at a distance using telecommunications technology — has expanded from a peripheral modality into an established component of routine practice in many health systems [1, 2]. The COVID-19 pandemic compressed a decade of anticipated diffusion into months [3], and post-pandemic uptake has been sustained in primary care, mental-health services, monitoring of chronic conditions, and certain emergency contexts (notably stroke care) [4, 5]. In Israel, where the present study was conducted, all four major Health Maintenance Organizations (HMOs) integrated digital and remote services rapidly during and after the pandemic, with subsequent expansion catalyzed further by the security situation that began in October 2023.

Despite this clinical reality, formal medical education on telemedicine remains uneven [6]. Although the case for integrating telemedicine into medical curricula was articulated as early as the work of Demiris on graduate medical informatics education [7], a growing literature documents both curricular gaps and a range of pedagogical responses [8], including dedicated electives, simulation-based modules, and integrated longitudinal exposure across pre-clinical and clinical years. Studies of student attitudes have generally relied on pre/post surveys [9] or on qualitative analysis of small focus groups [10]. Reflective writing has long been used as a pedagogical instrument in medical education [11] and is increasingly being analyzed as a data source for understanding student conceptual development on emerging clinical topics [12]. The pre-clinical years — in which students may not yet have direct clinical exposure to telemedicine — are arguably the years in which professional identity is most actively forming [13], yet they are under-represented in the published telemedicine-education literature.

What makes reflective writing a particularly well-suited instrument for studying technology learning in medicine is that engagement with new clinical technologies is rarely a purely cognitive event. The acquisition of telemedicine competencies involves not only factual knowledge and procedural skill but also a reorganization of the learner’s professional self-concept, a process that draws on both conscious appraisal and less-than-fully-conscious affective and identity-related responses [14]. Learning to use unfamiliar technologies in clinical contexts can mobilize anxieties about professional adequacy and about the boundaries of good care; it can also generate enthusiasm, identification, or ambivalence that reflects deeper assumptions about what medicine is and ought to be [15]. These internal processes: affective, identity-laden, and not always accessible to direct self-report are precisely those that reflective writing, when sustained over the course of a learning experience, is positioned to surface and render visible. The reflective diary, in particular, constitutes an extended first-person record in which learners articulate attitudinal shifts, biographical resonances, and emerging professional commitments in ways that survey instruments are structurally unable to capture [12]. This consideration was central to the methodological choice guiding the present study: rather than measuring telemedicine attitudes as a static variable, we sought to follow the texture of students’ conceptual and affective engagement as it unfolded across an entire elective course, and to analyze that engagement using an approach, reflexive thematic analysis, suited to the interpretive character of the data.

We adopt an andragogical framing for this study: our participants are adult learners (medical students in the pre-clinical years), and the reflective diary is understood as an instrument well suited to adult learners’ self-directed engagement with new material, their integration of prior experience, and their orientation toward immediate professional relevance [16]. This framing shapes our reading of the reflections and, in particular, our interpretation of the pedagogical themes (Sect. 4.2).

The multi-cohort design of the present study was motivated by three considerations. First, while single-cohort qualitative work on student telemedicine attitudes has been valuable, collecting reflections across two temporally distinct groups permits comparative attention to how student engagement is shaped by the surrounding socio-technical context. Second, the post-pandemic period has introduced new normatively-loaded considerations — most notably the rapid public emergence of generative artificial intelligence and an evolving debate about the boundaries of remote care — whose articulation in student reflections has not been systematically described. Third, comparing thematic patterns across cohorts allows us to describe (qualitatively) whether the taught curriculum elicits stable versus context-sensitive engagement; we make no quantitative or causal claim from these patterns.

The present study reports a reflexive thematic analysis of 81 reflective diaries from two cohorts of students (a retrospective cohort, 2019–2024, and a prospective cohort, 2025) enrolled in a single elective telemedicine course at one Israeli medical school. Our analytic objectives were: (a) to identify themes characterizing students’ substantive engagement with telemedicine in their reflective writing; (b) to examine the stability of the thematic structure across cohorts spanning the COVID-19 and post-pandemic periods; and (c) to describe cohort-specific differences in the articulation of themes that may reflect changes in the broader telemedicine discourse (without inferring causation).

## Methods

### Setting and Course

Data were drawn from an elective course on Telemedicine (“Telemedicine — Medicine in the Digital Age”, course no. 275304) offered at the Technion – Israel Institute of Technology, Faculty of Medicine. The course is taught by a single instructor (a pediatrician and active researcher in telemedicine), is delivered entirely via the Zoom video-conferencing platform (Thursdays, 17:30–19:30) during one semester (Spring) for two academic credits, with a minimum of 75% attendance required. Course content covers clinical, technological, ethical, legal, social, and economic dimensions of telemedicine, with attention to applications including telestroke, telepsychiatry, teleradiology, remote monitoring of chronic conditions, and telediagnostic devices, with comparative material drawn from different countries. The course also includes lectures on the difficulties physicians encounter in telemedicine, decision-making hurdles in telemedicine and tele-triage, safety considerations, and patient–physician relationships in remote care. The thirteen lecture topics were: (1) Introduction; (2) Uses of telemedicine; (3) Advantages and disadvantages; (4) Telemedicine in Israel; (5) Telemedicine internationally; (6) Legal and ethical aspects; (7) Social aspects; (8) Physicians’ challenges and lived experience in telemedicine; (9) Economic aspects; (10) Clinical decision-making in the telemedicine era; (11) Safety and tele-triage; (12) Doctor–patient relationships in the telemedicine era; and (13) A guest lecture on the patient’s perspective. The course does NOT include any clinical placement or hands-on telemedicine practicum; students who took part in the corpus had no other formal telemedicine instruction elsewhere in their curriculum. The course is open primarily to second-year medical students; first-year and third-year medical students, and students from other faculties (e.g., Computer Science), may enroll on special request. Typical annual enrollment is approximately 40–45 students. Course assessment is based on a written summary paper on a self-selected telemedicine-related topic (100% of the course grade); in addition, students are required to submit a reflective diary at the end of the semester as a mandatory course submission that is not graded. There were no specific requirements for the reflective diary. Diary instructions were deliberately open-ended: students were simply asked to write about the course — what they thought about it, its value for them, its pros and cons, and any suggestions for improvement. No structured prompt list, minimum word count, or set of guiding questions was provided, in keeping with the aim of eliciting spontaneous, biographically anchored reflection. The reflective diaries constitute the data corpus for the present study. 

### Sample and Cohort Structure

The corpus comprises 81 reflective diaries spanning two cohorts distinguished by the timing of consent: a retrospective cohort and a prospective cohort. Cohort 1 (retrospective, 2019–2024, *n* = 39) consists of diaries originally submitted at the end of the relevant academic year as a regular component of the course; consent for use of these diaries in qualitative research was obtained retrospectively in 2025: all former students whose diaries were retained were contacted by a single email round (in 2025, after ethical approval was granted) and asked to provide written consent. Cohort 2 (prospective, 2025, *n* = 42) consists of diaries submitted during the Spring 2025 course; students were informed at the beginning of the course that the diaries would be analyzed for research and were asked to enclose a signed consent form with their diary. Of the 81 participants, 79 were second-year medical students at the time of writing; one was a Computer Science student who took the course as a cross-faculty elective (Cohort 1), and one was a third-year medical student (Cohort 2). The class included Hebrew-speaking students of both Jewish-Israeli and Arab-Israeli backgrounds. Diary length varied from approximately 100 to 900 words (median ≈ 350 words). Sample characteristics by cohort are summarized in Table 1.

**Table 1:** Sample characteristics by cohort (*n* = 81)

Characteristic	Cohort 1 (retrospective, 2019–2024)	Cohort 2 (prospective, 2025)	Total
n	39	42	81
Year-2 medical students	38	41	79
Other program/year	1 (CS, cross-faculty)	1 (Year-3 medical)	2
Demographic mix	Jewish-Israeli, Arab-Israeli	Jewish-Israeli, Arab-Israeli	—
Submission window	End-of-semester, 2019 – early 2024	Jun – Aug 2025	—
Approx. diary length, words (range)	~ 330 (~ 100–890)	~ 370 (~ 110–910)	~ 350 (~ 100–910)
Timing of consent	Retrospective (by email in 2025, after ethical approval)	Prospective (at start of course; consent enclosed with diary)	81 / 81 consented

### Ethics and Consent

All 81 participants whose diaries are included in the analytic corpus provided written informed consent for qualitative research analysis under the protocol “Qualitative Evaluation of Medical Students’ Feedback in the ‘Telemedicine’ Course” (Principal Investigator: [name redacted for blinding]). The two cohorts differed in the timing and procedure of consent. For Cohort 2 (2025, n = 42), consent was sought prospectively: students were informed at the beginning of the course that their reflective diaries would be analyzed for research and were asked to enclose a signed consent form together with their submitted diary. For Cohort 1 (2019–2024, n = 39), diaries had been submitted at the end of the relevant course year as a routine course component, before any research framing was in place; consent was sought retrospectively in 2025: after ethical approval was granted (January 2025), all former students whose diaries had been retained were contacted by email in a single round and asked to provide written consent for use of their diaries in research. Only diaries from students who returned a signed consent were retained for analysis. In both cohorts, students were informed that participation in the research was voluntary, fully anonymized, and would have no effect on the course grade (which is determined separately from the diary). No participant was excluded from the analytic corpus on grounds other than non-consent.

The study received ethical approval from the Technion ethics committee, approval number 2025-003, dated 7 January 2025.

### Anonymization

All identifying information (names, national identification numbers, email addresses, distinctive geographic identifiers) was removed at the analytic stage. Each participant is referenced as P01–P81 throughout this manuscript. A researcher-only mapping table is retained securely and is not included in the supplementary materials.

### Analytic Approach

We followed Braun and Clarke’s six-phase reflexive thematic analysis [17, 18] with an inductive (data-driven) orientation. The unit of coding was the meaningful text segment (clause to paragraph). Codes were generated bottom-up across the full corpus. Cohort 1 was coded first, yielding an initial framework of 47 codes nested under 6 themes and 19 sub-themes. Cohort 2 diaries were then coded against this framework, with new sub-themes and codes added inductively where new ideas appeared and existing codes refined. The final framework comprised 91 codes nested under 6 themes and 29 sub-themes; the high-level theme structure remained stable across both cohorts, with sub-theme expansion concentrated in Theme 4 (notably the addition of breaking bad news, defensive medicine, and online patient/clinician communities), Theme 2 (artificial intelligence as next horizon), Theme 3 (engagement with the instructor’s own published research), and Theme 5 (pre-medical professional background). The themes are described in Table 2, while full framework is summarized in Table 3. Quotations selected for inclusion were chosen for representativeness of frequently coded patterns or, where explicitly noted, for distinctive divergent or counter-positions. All diaries were written in Hebrew; quotations were translated into English, with a second native Hebrew speaker checking translations against the originals. Coding was undertaken by two researchers (MH and IC). MH conducted the initial line-by-line coding across the full corpus and drafted the initial codebook (structured as themes, sub-themes, and codes). IC then independently reviewed a random sub-sample of approximately 20% of diaries using the emerging codebook, and IC and MH reconciled codes and sub-themes through iterative discussion until consensus was reached. Coding was carried out manually (Microsoft Word for annotation and Microsoft Excel for the codebook and Theme × Participant matrix); no dedicated qualitative-analysis software (e.g., NVivo, Atlas.ti, Dedoose) was used. Analytic credibility was supported by (a) constant comparison across codes and cohorts; (b) peer debriefing between the two coders throughout the analysis; (c) thick description of quoted excerpts in the Results; and (d) an explicit audit trail in the form of a full codebook with frequencies, provided as Supplementary Table S1. A formal inter-rater reliability statistic was not calculated; this is consistent with Braun and Clarke’s [18] guidance for reflexive thematic analysis, in which coding is treated as an interpretive act rather than as the retrieval of pre-existing categories, and inter-coder agreement is not the appropriate quality index. This choice is discussed further in § 4.6.

### Reflexivity Statement

The Zoom delivery format of the course is itself a recurring topic of reflection in the corpus, and the corpus is therefore both about telemedicine pedagogy and shaped by it. Diaries were submitted to the course instructor by name, which likely shapes the corpus toward favorable evaluations of teaching and toward expressed gratitude. We have addressed this courtesy bias in the limitations section. The two cohorts also differ in a methodologically consequential way: Cohort 1 wrote without knowledge that the diaries would later be analyzed for research, with consent obtained retrospectively, while Cohort 2 wrote knowing that the diaries would be analyzed and with consent in place from the outset. We do not assume that this difference is neutral and we comment on it explicitly where the two cohorts diverge in articulation. Consistent with best practice for reflexive research, we describe our positionality explicitly. MH is a board-certified pediatrician, pediatric hematologist, and an active researcher in telemedicine (with a PhD degree on this subject), and is the course instructor. This is a substantial positionality: MH holds detailed prior expectations about the taught content and about which themes are likely to arise, and the students knew throughout that MH would be reading their diaries. We acknowledge both the risk of confirmatory reading in the analysis and the courtesy-bias effect on the diaries themselves. IC is a researcher in health-services management at the College of Management Academic Studies, was not involved in the design, delivery, or assessment of the course, and had no prior relationship with any of the participants; IC therefore reviewed the data as an outsider to the course and its instructor. Both authors have prior experience with adult-learner research and with reflective writing as an educational method. The two-coder arrangement (initial coding by the insider, independent review by the outsider) was chosen deliberately as one counterweight to MH’s positionality; residual bias, however, cannot be excluded, and this is a limitation of the present analysis (Sect. 4.6).

### Trustworthiness

We have followed the Standards for Reporting Qualitative Research (SRQR) [19] in the present manuscript and have prepared the dataset and codebook in a form compatible with the Consolidated Criteria for Reporting Qualitative Research (COREQ) [20]; a completed checklist is available as supplementary material. To support transferability, the codebook with frequency counts (Supplementary Table S1) and a Theme × Participant matrix (Supplementary Table S2) are provided in the accompanying spreadsheet.

## Results

Six themes were identified, organized into 29 sub-themes. Approximate theme-level prevalence (number of diaries in which the theme was present, of *n* = 81) is given in Table 2; the full theme and sub-theme structure is given in Table 3 below. Full code-level frequencies are provided as Supplementary Table S1. Themes 1–4 form the substantive intellectual content of the reflections; Theme 5 is selectively triggered by individual biography; Theme 6 is near-universal because pedagogy is consistently commented on.

For orientation, the six themes correspond broadly to elements of the taught syllabus. Theme 3 (a holistic discipline: multi-dimensional engagement) reflects the multi-topic structure of the 13 lectures (see § 2.1). Theme 4 (equity, access, and the doctor–patient bond) is anchored in the lectures on social aspects (lecture 7), legal and ethical aspects (lecture 6), and doctor–patient relationships (lecture 12). Theme 2 (telemedicine as telos) is a synthetic reading of the cross-cutting lectures on international and Israeli telemedicine (lectures 4–5). Theme 1 (transformative conceptual journey), Theme 5 (lived experience as interpretive lens), and the pedagogical-resonance observation within Theme 6 were NOT taught topics; they arose spontaneously in students’ writing and are, in that sense, the more distinctive contributions of the reflective-diary instrument used here.

**Table 2:** Themes identified in the reflexive thematic analysis (*n* = 81)

#	Theme	Prevalence *n* (%)
1	A transformative conceptual journey: from naïveté or skepticism to conviction	59 / 81 (73%)
2	Telemedicine as telos: future-orientation and professional identity	67 / 81 (83%)
3	A holistic discipline: multi-dimensional critical engagement	57 / 81 (70%)
4	Equity, access, and the doctor-patient bond: a productive tension	59 / 81 (73%)
5	Lived experience as interpretive lens	34 / 81 (42%)
6	Pedagogy and the medium: form reflecting content	77 / 81 (95%)

The final analytic framework is described here in Table 3.

Sub-themes marked with * are framework additions made during coding of Cohort 2 diaries; the high-level six-theme structure was preserved unchanged from the initial framework developed during coding of Cohort 1. Brief characterizations are intended as orientation rather than as exhaustive code-level descriptions; readers requiring code-level detail are referred to Supplementary Table S1.

**Table 3:** Final analytic framework: themes and sub-themes (*n* = 81)

Theme	Sub-theme	Brief characterization
1. A transformative conceptual journey: from naïveté or skepticism to conviction	1.1 The starting position	Active skepticism, narrow conception (TM as ‘an app’), or curiosity without prior substantive knowledge
	1.2 The expansion moment	Cumulative realization of the field’s breadth across multiple lectures, rather than a single ‘turn’
	1.3 From private revisions to public advocacy	Sharing learning with family/peers; recommending the course; advocating for its conversion to mandatory status
2. Telemedicine as telos: future-orientation and professional identity	2.1 Inevitability of digital medicine	Telemedicine framed as the future of the profession, asserted with little hedging
	2.2 COVID-19 — and now, war — as catalysts	Pandemic and (in Cohort 2) the October 2023 war framed as step-change events legitimizing telemedicine
	2.3 Specialty-specific reflection	Naming concrete specialties (telestroke, telepsychiatry, pediatric oncology, smartwatch cardiology, teleradiology)
	2.4 AI as next horizon *	AI articulated as the next stage beyond current telemedicine: both as promise and as source of existential discomfort
3. A holistic discipline: multi-dimensional critical engagement	3.1 The ‘many lenses’ recognition	Recurrent enumeration of perspectives: economic, ethical, legal, social, technological, doctor–patient
	3.2 Pre-clinical curriculum gap	Course experienced as filling a gap, often framed as ‘macro vs micro’ relative to the rest of the curriculum
	3.3 Topical density and named standout topics	Decision-making (Kahneman/Tversky), ethics, telestroke, EMR; specific lectures named as memorable
	3.4 Engagement with instructor’s published research *	Direct citation of the instructor’s own published work as evidence within student reflections
4. Equity, access, and the doctor–patient bond: a productive tension	4.1 The equity promise	Geographic accessibility, reduced waiting times, cost-efficiency, specialist-to-specialist consultation
	4.2 The digital-divide paradox	Populations most needing telemedicine (elderly, low-SES, disabled) least equipped to use it
	4.3 The doctor–patient bond: erosion vs. augmentation	Both positions articulated, often within a single diary; the corpus’s most thoughtful disagreement
	4.4 Loss of physical examination	Concrete clinical concern about the limits of remote assessment and diagnostic accuracy
	4.5 Breaking bad news and high-stakes encounters *	Boundary case for technological substitution; affective limits of remote care articulated
	4.6 Defensive medicine in remote care *	Defensive prescribing under remote uncertainty; patient-side moral hazard from increased availability
	4.7 Online patient and clinician communities *	Social-media health communities as a parallel digital-health phenomenon (singleton observation, P80)
5. Lived experience as interpretive lens	5.1 Self as patient	Personal use of HMO digital services (Maccabi, Klalit) as foundational interpretive resource
	5.2 Self or family member as chronic patient	Diary tone shifts toward concern about erosion of bond for those most dependent on it
	5.3 Family illness and high-affect biographical moments	Major family medical events (parental illness, pregnancy under COVID) framing engagement
	5.4 Geography and global health	Peripheral residence; volunteer work in low-resource settings; rural-medicine framing
	5.5 Pre-medical professional and prior tech background *	Medical-secretary work, national service in clinical wards, military intelligence-corps tech roles
6. Pedagogy and the medium: form reflecting content	6.1 Zoom as enacted argument	Delivery format read as demonstrating the course’s content (the proposed ‘pedagogical resonance’)
	6.2 The teacher	Near-universal warm references; calm, patient, flexible, attentive; expertise recognized in Cohort 2
	6.3 Pedagogical scaffolding	Embedded videos, physical telemedicine devices, guest lectures; integration with Health Economics
	6.4 The reflective paper as deeper learning	Paper-over-exam appreciated; freedom to choose biographically resonant topics valued
	6.5 Logistical tensions and constructive critique	Scheduling, lecture overlap, course overlap; one Cohort-C Zoom-skeptic counter-voice (P49)
	6.6 Suggestions	Hands-on simulations, more guests, patient voices, AI content, breakout rooms, mandatory conversion

Sub-themes marked with * were expressed only in Cohort 2 (2025) and were absent or rare in Cohort 1’s reflections

### Theme 1 — A Transformative Conceptual Journey

Approximately three-quarters of participants framed the course as a journey of attitudinal change, consistent across the two cohorts. Three starting positions recurred: active skepticism or aversion (often framed in terms of the loss of human contact); narrow conception (typically equating telemedicine with consumer health applications); and curiosity without prior substantive knowledge.

*“When I registered for the course*,* and already in the first lecture*,* I thought to myself: what is this for? Why push this field of remote communication into medicine?”* (P38, Cohort 1).

*“Before the course began I had a very narrow view of what telemedicine is. I thought of a phone call I had with a doctor a few months ago*,* in which I felt mainly insecurity and a lack of connection. I did not imagine that an entire field stood behind this.”* (P73, Cohort 2).

The transformative arc was rarely tied to a single lecture; instead, students described a cumulative realization of breadth. A distinctive sub-theme was the move from private revision of view to public advocacy: students described sharing what they had learned with family and recommending the course to others. A specific position appearing in both cohorts but markedly more frequent in Cohort 2 was the recommendation that the course be converted from an elective to a mandatory component of the curriculum.

*“I see this course as a foundational course for every medical student*,* and in my opinion it should be included as part of the basic curriculum and not remain an elective.”* (P71, Cohort 2).

### Theme 2 — Telemedicine as Telos

Where Theme 1 was autobiographical, Theme 2 was prospective: telemedicine was positioned as the inevitable trajectory of medicine. This framing was the most uniformly stated proposition in the corpus, asserted with little hedging.

*“Telemedicine is the future of the health world*,* and the COVID-19 pandemic proves this beautifully.”* (P20, Cohort 1).

*“Already today telemedicine is an inseparable part of medicine*,* and its importance will only grow.”* (P65, Cohort 2).

Cohort 1 participants frequently invoked COVID-19 as a step-change event. Cohort 2 extended this catalyzing-event narrative to include the war:

*“Until a few years ago this field was hardly discussed or emphasized in medical studies*,* but in light of the reality we experienced during COVID and the war*,* I understood how essential it is for ensuring continuity of care and patient access.”* (P70, Cohort 2).

*“Although it was not under our control*,* the fact that the course was delivered during wartime illustrated just how relevant telemedicine is to daily life. Between the lectures I learned of relatives’ experiences with telemedicine*,* and against the backdrop of the war many news stories and campaigns on the topic appeared.”* (P80, Cohort 2).

A noteworthy distributional shift was the appearance, in Cohort 2 only, of explicit articulations of artificial intelligence as the next horizon — sometimes in optimistic terms, sometimes as a source of existential discomfort:

*“When will the time come when patients speak with AI and not with a human medical figure of flesh and blood like themselves? When will telemedicine enter the field of delivering bad news*,* where a patient will receive bad news through a doctor on a screen? It seems the day is not far off*,* and one can say this is actually a slightly threatening thought.”* (P73, Cohort 2).

Specialty-named reflection — students naming concrete future specialties in which they imagined telemedicine playing a role — was substantially more frequent in Cohort 2, with telepsychiatry, pediatric oncology, smartwatch-based cardiology, and teleradiology each named by distinct participants in their reflections on chosen paper topics.

### Theme 3 — A Holistic Discipline

Theme 3 captured students’ substantive intellectual gains. A near-formulaic phrase appeared across many diaries: “from different angles” or “from various aspects”, typically followed by an enumeration of the perspectives the course brought to bear:

*“We approached the field of telemedicine from many directions: economic*,* ethical*,* legal*,* doctor-patient relations.”* (P04, Cohort 1).

*“The course exposes us to things we don’t get to engage with in any other course and gives a view into a whole field of medicine that gets less emphasis.”* (P61, Cohort 2).

Several participants explicitly framed this multi-dimensional engagement as filling a gap in their pre-clinical curriculum, with one Cohort 1 participant invoking a macro-versus-micro framing: “in our studies we deal a lot with the micro of medicine, but this course in its essence deals with the macro” (P08). The single most frequently named standout topic in Cohort 2 was the lecture on clinical decision-making, sometimes occasioning extended reflections engaging dual-process accounts and Kahneman and Tversky’s work on subjective probability and heuristics (P69, P74). A small but distinct sub-theme appearing in Cohort 2 was direct engagement with the instructor’s own published research as evidence within student reflections (P51).

A late addition to the named-standout list, present in Cohort 2 only, was the electronic medical record (EMR) and its impact on clinical decision-making, transparency between physician and patient, and documentation processes:

*“We learned about the computerized medical record*,* and the way in which it influences the doctor’s decision-making*,* transparency vis-à-vis the patient*,* and the process of documentation and oversight — a field I had not encountered in depth in previous courses.”* (P79, Cohort 2).

### Theme 4 — Equity, Access, and the Doctor-Patient Bond

Theme 4 captured the substantive intellectual center of the course in students’ accounts: a productive tension between telemedicine’s equity promise and its costs to physical presence and the therapeutic relationship. Most participants identified geographic accessibility, reduction of waiting times, and economic efficiency as primary equity gains. A digital-divide paradox — that the populations telemedicine ought to help most are those least equipped to use it — was articulated repeatedly:

*“On the one hand*,* accessibility for people from remote areas… On the other hand*,* the weak populations*,* the elderly and residents of developing countries*,* are precisely those who have difficulty obtaining access to computing services for economic reasons and difficulties understanding the technology.”* (P03, Cohort 1).

Sub-theme 4.3 contained the corpus’s most thoughtful disagreement, with students articulating both an erosion position (telemedicine corrodes the therapeutic relationship) and an augmentation position (telemedicine strengthens it through increased frequency and accessibility). Many held both positions in tension within a single diary. A particularly clear augmentation case was articulated by a Cohort 2 participant who reported that an actual telemedicine encounter had felt more attentive than an in-person encounter:

*“I had a personal experience in which precisely in an online encounter there was a sense of being listened to*,* and room for conversation*,* even more than in a face-to-face encounter.”* (P80, Cohort 2).

Two sub-themes emerged for the first time in Cohort 2 and warrant separate description.

First, breaking bad news and other high-affect encounters were articulated as a boundary case for technological substitution. The most extended treatment, from a single Cohort 2 participant, framed this as a fundamental humanistic limit:

*“Personally I find it hard to accept a situation in which a doctor would tell me about the death of a close person or about a serious illness like cancer through a screen… There are cases that require full human presence — eye contact*,* facial expressions*,* gestures of compassion — things that*,* sadly*,* disappear in the move to telemedicine. Otherwise*,* what is the difference between us and a robot?”* (P73, Cohort 2).

Second, two Cohort 2 participants articulated specific economic-clinical concerns about behavioral responses to remote care. One named defensive prescribing as a likely consequence of clinician uncertainty under remote conditions:

*“[There is also] the lack of confidence and the fear the doctor feels in making a decision that may be fateful for the patient — and consequently the prescribing of medicines unnecessarily*,* which may cause harm*,* in the framework of the concept of cover your ass.”* (P73, Cohort 2).

Another named patient-side moral hazard from increased availability (P61, Cohort 2). These second-order economic-clinical concerns were largely absent from the Cohort 1 corpus.

A further emerging sub-theme — articulated by only a single participant in the present corpus but representing a conceptually distinct dimension of digital medicine — was the role of online patient and clinician communities on social media platforms. The participant framed these communities as a phenomenon parallel to formal telemedicine services, with their own affordances and risks:

*“Patient and physician communities on social networks are an amazing phenomenon that holds within it much information and support but also challenges of reliability and boundaries. In an era when so much information flows online*,* it is important to know how to filter*,* analyze*,* and use this tool responsibly. We need to know how to accompany patients there too*,* or at least to understand how it affects them.”* (P80, Cohort 2).

This is a singleton observation in the present corpus, and we do not over-interpret it; it is reported here because it represents a conceptually distinct dimension of digital medicine that is increasingly recognized in the broader literature on e-patients and online health information [21] and that may emerge more prominently in subsequent cohorts.

### Theme 5 — Lived Experience as Interpretive Lens

Approximately a third of all diaries (34 / 80) contained a personal-biographical episode the writer used explicitly to organize their engagement with the course. The most common biographical resource was the writer’s own use of telemedicine services as a patient. Variants included the writer or a close family member as a chronic patient (P61, P62, both Cohort 2); a family member’s serious illness (P27, Cohort 1 — the writer described “closing a circle” with respect to her father’s illness); pregnancy under COVID-19 conditions (P23, Cohort 1); residence in a peripheral area with restricted clinic access (P10, Cohort 1); volunteer work in a low-resource setting (P05, Cohort 1 — Uganda); a prior technological or military background (P04, P21, Cohort 1); and pre-medical clinical roles, a biographical lens that appeared exclusively in Cohort 2 (P77 had worked as a doctor’s secretary; P75 had completed national service in an internal-medicine ward).

*“Few courses have made me feel I closed a circle with what I experienced and felt when my father was sick. There is nothing more satisfying than the opportunity you gave me to meet the topic from another angle.”* (P27, Cohort 1).

Theme 5 interacted with all other themes: students with strong biographical engagement produced substantively richer Theme 4 reflections than those without. The pattern was stable across Cohort 1 and Cohort 2.

### Theme 6 — Pedagogy and the Medium

The most universally commented-on aspect of the course was its pedagogy. The most distinctive observation — phrased almost epigrammatically and recurring in nearly identical form across many independently-written diaries — was that the Zoom delivery format was experienced as enacting the course’s argument:

*“Even the fact that the course is delivered on Zoom — very fitting in my eyes and reinforces the underlying idea of telemedicine. From the understanding that remote work works.”* (P08, Cohort 1).

*“The Zoom mode of learning corresponded beautifully with the essence of the course. We saw how Zoom enables learning and communication*,* and we even experienced the difficulties that may arise in using telemedicine — for example in the lesson where the lecturer’s computer didn’t work at first.”* (P65, Cohort 2).

That this argument appeared in essentially the same form across many independently written diaries- suggests it is an inference students arrive at almost inevitably under the conditions of the course. P65’s gloss is particularly noteworthy: a Zoom failure during class was reframed by the student as itself a lesson in telemedicine’s affordances and limits. A single but distinct counter-voice complicates the otherwise consistent finding:

*“Already from the first lessons I felt that despite the comfort of learning from home*,* I personally find it harder to connect to lectures in this format. The feeling of distance from the lecturer and from the other students sometimes made me feel less involved and less emotionally present in the learning.”* (P49, Cohort 2).

References to the instructor were nearly universal and consistently warm. Cohort 2 participants more frequently named the instructor’s clinical and research expertise; this is consistent with Cohort 2’s distinctive engagement with the instructor’s own published work. Three pedagogical features were repeatedly named as effective: embedded videos; physical telemedicine devices brought to virtual class (Cohort 1 principally); and guest lectures. Constructive suggestions volunteered by participants included hands-on simulation components (most frequent across both cohorts), more guest lecturers, patient voices brought into the course, more content on artificial intelligence and on telepsychiatry, breakout-room discussions in Zoom (Cohort 2), and integration with the Year-1 / Year-3 clinical-exposure courses already in the curriculum (Cohort 2). The recommendation that the course be made mandatory was present in Cohort 1 and substantially more frequent in Cohort 2.

### Cross-Cutting Findings

#### Thematic Stability Across Cohorts

The thematic structure derived from Cohort 1 did not require fundamental restructuring when Cohort 2 diaries were added; new codes were added inductively but no new themes or sub-themes at the highest level. We interpret this as supporting substantial thematic stability across cohorts spanning the late-pandemic and post-pandemic periods.

#### Cohort Drift in Distribution and Articulation

Despite stability of themes, several distributional shifts were visible across cohorts. The mandatory-course recommendation was markedly more frequent in Cohort 2. Specialty-specific reflections (telepsychiatry, pediatric oncology, smartwatch cardiology, teleradiology) clustered in Cohort 2. AI-as-next-horizon and the breaking-bad-news boundary case were largely Cohort 2 phenomena. The war as a catalyzing event appeared only in Cohort 2. The electronic medical record and online patient communities entered the corpus only in Cohort 2. We interpret these shifts as reflecting changes in the broader public discourse on telemedicine, AI, and digital health between the cohorts, rather than changes in course content. Table 4 reports cohort-specific prevalence for the sub-themes that show meaningful drift. We emphasize that these are qualitative differences between two cross-sectional cohorts, not measures of temporal change. Because the two cohorts differ not only in year but also in consent procedure (retrospective versus prospective), we cannot rule out selection or writing-context effects. We use the term ‘difference in articulation’ rather than ‘shift’, ‘drift’ or ‘emergence’ where a temporal or causal reading might be inferred; the taught curriculum did not change substantively between the two cohorts, so the differences reported here are unlikely to be explained by course content.

**Table 4:** Sub-themes showing meaningful differences in expression between Cohort 1 (retrospective, *n* = 39) and Cohort 2 (prospective, *n* = 42). Counts and rounded percentages are based on binary presence/absence coding at the sub-theme level

Sub-theme	Cohort 1 *n* (%)	Cohort 2 *n* (%)	Direction
1.3d Course should be made mandatory	2 (5%)	7 (17%)	▲
2.2b War / national emergency as catalyzing event	0 (0%)	4 (10%)	▲
2.4 AI as next horizon (combined: promise + displacement)	1 (3%)	6 (14%)	▲
3.3c Decision-making lecture as standout topic	2 (5%)	6 (14%)	▲
3.3e Electronic medical record as standout topic	0 (0%)	1 (2%)	▲ (new)
3.4 Citing instructor’s published research	0 (0%)	3 (7%)	▲ (new)
4.5 Breaking bad news as boundary case	0 (0%)	3 (7%)	▲ (new)
4.6 Defensive medicine / moral hazard concerns	0 (0%)	2 (5%)	▲ (new)
4.7 Online patient/clinician communities	0 (0%)	1 (2%)	▲ (new)
5.5c Pre-medical clinical work as biographical lens	0 (0%)	2 (5%)	▲ (new)
6.1d Zoom-skeptic counter-voice	0 (0%)	1 (2%)	▲ (new)
6.6 h Breakout rooms / small-group suggestion	0 (0%)	1 (2%)	▲ (new)
6.6i Integration with Year-1/3 clinical exposure	0 (0%)	3 (7%)	▲ (new)
6.6j More telepsychiatry / mental-health content	0 (0%)	2 (5%)	▲ (new)

All shifts in Table 4 are in the direction of greater Cohort 2 prevalence; we found no sub-theme that was meaningfully more prevalent in Cohort 1. Counts are based on the codebook’s binary presence/absence coding at the sub-theme level. Percentages are rounded.

#### Courtesy Bias and Length-as-Depth

The corpus exhibits very few critical or negative reflections at the level of overall course evaluation. Specific frictions are named (scheduling, lecture overlap, content overlap with the instructor’s other course in Cohort 2) but always within a frame of overall appreciation. The introduction of formal research consent in Cohort 2 did not appear to substantially alter this distribution. Diary length varied substantially; shorter diaries tended to confine themselves to Theme 6 and a brief gesture toward Theme 2, while longer diaries (e.g., P06, P12, P23, P27, P37, P61, P69, P73, P77, P80) engaged Themes 3 and 4 substantively and frequently included Theme 5 biography. Length was an imperfect proxy for analytic depth and should not be treated as a measure of authenticity. Following reviewer feedback, we note that diary length is reported as context only and is not treated as an analytic variable. Consistent with reflexive-thematic-analysis practice, richness of engagement rather than word count was the criterion for illustrative quotation.

#### Demographic and Disciplinary Robustness

The class was ethnically and linguistically diverse (Hebrew-speaking and Arab-Israeli students); included one Computer Science student and one third-year medical student. Substantive themes were not differentiated by group: Arab-Israeli students’ diaries were not thematically distinct from their Jewish-Israeli peers’ on the substantive themes, and the cross-disciplinary participants confirmed rather than complicated the medical-student themes. We interpret this robustness across disciplinary, linguistic, and demographic backgrounds as itself a finding of interest.

## Discussion

This corpus illustrates how an elective telemedicine course functions in students’ reflective accounts as more than content delivery: as a site of attitudinal transformation, professional-identity formation, and integration of personal biography into emerging clinical thought. Several findings are of interest to the broader literature on telemedicine in medical education.

### The Pre-Clinical Curricular Role

Students value the course’s macro lens precisely because their pre-clinical curriculum is, in their own framing, micro. Telemedicine teaching in the pre-clinical years may be doing double duty: both teaching the discipline of telemedicine itself and providing a meta-reflective space on the practice of medicine more broadly. This is a pedagogically valuable function not always articulated in curricular planning [22]. Designers of telemedicine curricula should consider whether parallel courses (medical ethics, health economics, communication) play a similar dual function, and whether the macro-reflective space might be deliberately strengthened.

### Form-Content Alignment

The most distinctive pedagogical finding in our corpus is that the Zoom delivery is consistently read by students as enacting the course’s argument. We propose the term “pedagogical resonance” to describe this effect, in which the medium of instruction provides an experiential analogue to the substantive content being taught. The recurrence of this observation across many independently-written diaries, in essentially the same form, suggests that pedagogical resonance is not a marginal feature but a near-universal interpretive frame for these students. The Cohort 2 minority counter-voice (P49) is a useful corrective: pedagogical resonance is not unanimous, and instructors should not assume that students for whom remote learning is more difficult experience the form-content alignment as a benefit. To our knowledge, this effect has not been previously named in the digital-medical-education literature. We note that our learners are adults (Knowles [16]), and the observed pedagogical resonance can be read within andragogical principles as an instance of experiential learning: the medium of instruction becomes itself a piece of the content to be understood. This adult-learner framing is particularly apt for medical students in the pre-clinical years, who are professionally socializing while formally learning.

### Lived Experience as a Feature, not a Bias

Students did not bracket personal experience as a confound; they used it as an organizing frame. Reflective-diary instruments in medical education research should not treat biographical content as a methodological problem to be controlled but as part of how clinical thought is actually formed in early-career students. This is consistent with established findings in the reflective-practice literature [11] and with prior diary-based research from the same medical faculty, which identified “linking current experiences to past experiences” and “future perspective taking” as central mechanisms by which medical students construct professional identity [23]. Our Theme 5 (lived experience as interpretive lens) and Theme 2 (telemedicine as telos / future-orientation) appear to map onto these previously described mechanisms, suggesting that the constructive cognitive processes by which pre-clinical students engage with telemedicine are continuous with those by which they engage with the medical profession more broadly. The expansion of biographical lenses into pre-medical professional roles in Cohort 2 — medical-secretary work, national service in clinical wards — may reflect the specific demographic profile of contemporary Israeli medical students, many of whom enter medical school after several years of national service or other employment.

### Cohort Drift in Normatively Loaded Terrain

The shifts across cohorts — toward AI-displacement concerns, mandatory-course advocacy, the war as catalytic event, concrete specialty-specific reflection, attention to electronic medical records, and the emergence of online patient communities as a distinct dimension — are themselves a research finding. Medical-education research on telemedicine attitudes has often relied on cross-sectional measurement at single time points; our analysis suggests that what is measured in 2023 will not be exactly what is measured in 2025, even when course content remains stable. The articulation of breaking-bad-news as a boundary case for telemedicine, which appeared only in Cohort 2, is a particularly significant development: it represents the emergence in student discourse of a humanistic limit on technological substitution, and it appears to be linked in students’ minds with the broader question of AI displacement (P73’s reflection co-articulates these concerns). The singleton observation on online patient communities (P80) likewise points to a dimension of digital health that may demand attention in future cohorts as social-media-mediated peer support and information-seeking become more prominent in patient experience. We stress that the qualitative differences described here are cross-sectional. They do not constitute evidence of longitudinal change in students’ attitudes, of a causal effect of external events (AI, war), or of a change in the pedagogical value of the course. What they do suggest is that the topics students choose to articulate in reflective writing on telemedicine are context-sensitive, and that a snapshot taken in one year should not be treated as a durable characterization of student engagement with the field.

### The Curricular Question

The substantial increase in Cohort 2 of recommendations that the course be converted from an elective to a curricular requirement is not, in itself, evidence that conversion is warranted; reflective-diary writers may overstate the importance of courses they are currently enrolled in. However, when read alongside the findings on pre-clinical curricular role, pedagogical resonance, and the absence of equivalent macro-lens content elsewhere in the curriculum, the recommendation reflects a coherent student-perspective argument. We do not advocate a specific curricular policy but note that the question deserves engagement at the level of curricular committees, particularly given the documented expansion of telemedicine in Israeli clinical practice.

### Limitations

The study has several limitations. The corpus is from a single institution and a single instructor, limiting transferability. All diaries were written in Hebrew; English-language quotation involves translation choices that may have shifted nuance, although a second native Hebrew speaker independently checked translations. The two cohorts were collected under different consent procedures: Cohort 2 wrote knowing that the diaries would be analyzed for research, while Cohort 1 wrote without that knowledge and consented retrospectively. We cannot rule out that the prospective Cohort 2 wrote in a more deliberate or self-aware register; conversely, retrospective consent in Cohort 1 means that the texts reflect more spontaneous reflection but at the cost of some ethical complexity. Diaries were submitted by name to the course instructor as a required end-of-semester submission (the course grade is based on a separate written paper, not on the diary), introducing courtesy bias in both cohorts. Reflective writing is a self-report instrument and reflects what students choose to articulate, not the totality of their attitudes. Inter-rater coding was not performed in the present analysis; this is a methodological limitation that we plan to address in subsequent work by applying the codebook to a stratified subsample. Additional limitations require explicit acknowledgement. First, because the course is an elective, all participants self-selected into it, and the corpus therefore reflects students with pre-existing interest in telemedicine; findings cannot be generalized to the broader medical-student population. Second, the course provides theoretical instruction only (no clinical placement), so students’ reflections are on taught content and on their own biographical experience as patients or family members rather than on hands-on clinical practice; results may differ where telemedicine is taught alongside supervised clinical exposure. Third, a formal inter-rater reliability statistic was not calculated. As set out in § 2.5, this reflects our commitment to Braun and Clarke’s [18] reflexive-thematic-analysis tradition, in which inter-rater reliability is not the appropriate quality index; we rely instead on independent review of a coded sub-sample, iterative reconciliation, and an explicit audit trail. Fourth, the course instructor (MH) is one of the authors and was the coder of first pass. The two-coder arrangement with an independent outsider (IC) was intended as one counterweight, but residual confirmation bias cannot be excluded.

### Implications for Practice

Three implications follow. First, programs designing telemedicine instruction in the pre-clinical years should consider matching the medium of delivery to the content where feasible, while remaining attentive to students for whom remote delivery is a pedagogical hindrance rather than an enactment. Second, scaffolding reflective writing assignments to invite biographical engagement may deepen substantive engagement with the material; this need not be at the expense of analytic rigor. Third, telemedicine curricula should consider including content on the boundary cases of remote care — breaking bad news, end-of-life conversations, defensive prescribing under uncertainty, and increasingly the management of patients’ use of online health communities — as these are emerging concerns in student discourse and deserve formal pedagogical engagement rather than being left to spontaneous student articulation. More broadly, our findings align with recent competency frameworks calling for the systematic integration of telemedicine into undergraduate medical curricula [24], and with recent evidence for the feasibility and acceptability of multi-institutional telemedicine curricula for medical students [25].

## Conclusions

In this qualitative reflexive thematic analysis of 81 reflective diaries from medical students enrolled in an elective telemedicine course across two cohorts (retrospective, 2019–2024; prospective, 2025), we identified six themes that were stable across cohorts: a transformative conceptual journey from skepticism to conviction; telemedicine as the perceived future of the profession; recognition of telemedicine as a multi-dimensional discipline; a productive tension between equity gains and erosion of the doctor–patient bond; lived experience as an explicit interpretive lens; and pedagogy and the medium of instruction. Students consistently described the course as a formative and highly valuable experience, reporting meaningful attitudinal change, engagement with multiple substantive dimensions of telemedicine, and a sense that the material was directly relevant to their emerging professional identity. Many recommended that the course be made a mandatory part of the curriculum. Qualitative differences in the articulation of certain sub-themes — notably AI-displacement concerns, breaking bad news as a boundary case, attention to electronic medical records, and emerging interest in online patient communities — were more prominent in the prospective (2025) cohort, reflecting the evolving discourse on digital medicine. Taken together, our findings make a strong case for the inclusion of dedicated telemedicine instruction as a core component of the pre-clinical medical curriculum, both to prepare future physicians for a clinical reality that is already digital and to provide a valuable macro-lens on the profession that is otherwise scarce in the pre-clinical years. Pedagogical resonance — the alignment between the Zoom delivery format and the substantive content on remote care — emerges as a distinctive asset of this course design and one that curricular committees may wish to preserve when planning telemedicine instruction more broadly. While causal claims about course effectiveness lie beyond the scope of a qualitative study, the depth, breadth, and consistency of positive student engagement documented here support the genuine educational value of the course and its wider adoption.

## Supplementary Information

Supplementary Material 1.

Supplementary Material 2.

## Data Availability

The full codebook with code-level frequencies and the Theme × Participant matrix are provided as Supplementary Tables S1 and S2. Anonymized excerpts beyond those reproduced in the manuscript are available from the corresponding author upon reasonable request, subject to ethics approval. Original Hebrew diaries are not publicly available in order to protect participant anonymity.
